# *De Novo* Transcriptome Analysis of Wing Development-Related Signaling Pathways in *Locusta migratoria* Manilensis and *Ostrinia furnacalis* (Guenée)

**DOI:** 10.1371/journal.pone.0106770

**Published:** 2014-09-10

**Authors:** Suning Liu, Wei Wei, Yuan Chu, Long Zhang, Jie Shen, Chunju An

**Affiliations:** Department of Entomology, College of Agriculture and Biotechnology, China Agricultural University, Beijing, China; Kansas State University, United States of America

## Abstract

**Background:**

Orthopteran migratory locust, *Locusta migratoria*, and lepidopteran Asian corn borer, *Ostrinia furnacalis*, are two types of insects undergoing incomplete and complete metamorphosis, respectively. Identification of candidate genes regulating wing development in these two insects would provide insights into the further study about the molecular mechanisms controlling metamorphosis development. We have sequenced the transcriptome of *O. furnacalis* larvae previously. Here we sequenced and characterized the transcriptome of *L. migratoria* wing discs with special emphasis on wing development-related signaling pathways.

**Methodology/Principal Findings:**

Illumina Hiseq2000 was used to sequence 8.38 Gb of the transcriptome from dissected nymphal wing discs. *De novo* assembly generated 91,907 unigenes with mean length of 610 nt. All unigenes were searched against five databases including Nt, Nr, Swiss-Prot, COG, and KEGG for annotations using blastn or blastx algorithm with an cut-off E-value of 10^−5^. A total of 23,359 (25.4%) unigenes have homologs within at least one database. Based on sequence similarity to homologs known to regulate *Drosophila melanogaster* wing development, we identified 50 and 46 potential wing development-related unigenes from *L. migratoria* and *O. furnacalis* transcriptome, respectively. The identified unigenes encode putative orthologs for nearly all components of the Hedgehog (Hh), Decapentaplegic (Dpp), Notch (N), and Wingless (Wg) signaling pathways, which are essential for growth and pattern formation during wing development. We investigated the expression profiles of the component genes involved in these signaling pathways in forewings and hind wings of *L. migratoria* and *O. furnacalis*. The results revealed the tested genes had different expression patterns in two insects.

**Conclusions/Significance:**

This study provides the comprehensive sequence resource of the wing development-related signaling pathways of *L. migratoria*. The obtained data gives an insight into better understanding the molecular mechanisms involved in the wing development in *L. migratoria* and *O. furnacalis*, two insect species with different metamorphosis types.

## Introduction

Insects are the only group of invertebrates that have evolved flight [1]. Their wings serve not only as organs of flight, but also may be adapted variously as protective covers [2], thermal collectors [3], gyroscopic stabilizers [4], sound producers [5], or visual cues for species recognition and sexual contact [6]. For those insects with wings during the adult stage, complete wings are not always visible throughout the life cycle. The insects undergoing incomplete metamorphosis have represented functional wings during the stage of nymph [7], while the insects going through complete metamorphosis only have wing discs inside the body in the larval stage [7]. Therefore, the comparative studies on how the insect wings are developed will be helpful to understand the insect metamorphosis. Additionally, given the high evolutionary conservation of the proteins involved in the wing development, the understanding of the molecular mechanisms involved in wing development also sheds light on organogenesis, tissue homeostasis, human disease and so on [8]–[11].

Insect wing development is controlled with amazing precision and complication. Current understanding of insect wing development mechanisms is mainly from the fruit fly *Drosophila melanogaster* [12]–[14]. The adult wing of fruit fly is derived from the wing imaginal disc, formed at the end of embryonic development [15]. In *Drosophila*, the wing imaginal disc is subdivided into anterior (A) and posterior (P) compartments at very early stage and then further subdivided into dorsal (D) and ventral (V) compartments at second instar stage [16]–[18]. Organizers located in the A/P and D/V boundaries coordinate the patterning of the wing disc by secreting signal molecules including the long-range morphogens Decapentaplegic (Dpp; the vertebrate homolog of which is TGFβ) and Wingless (Wg; the vertebrate homolog of which is Wnt) [11], [19], [20], and short-range morphogen Hedgehog (Hh) [21]. The produced morphogens form gradients to regulate the expression of target genes and control all aspects of wing development at cell level via the specific signaling pathways [19], [22]–[27]. These signaling pathways are highly evolutionarily conserved in many different animals. Some key genes for the wing disc development have also been identified in a few limited insect species, such as *Tribolium castaneum* [28]. However, knowledge about the identification and involvement of wing development-related genes in various insects, especially in non-model insects, is still unclear and incomplete.

Orthopteran migratory locust, *Locusta migratoria* manilensis, and lepidopteran Asian corn borer, *Ostrinia furnacalis* (Guenée), are two different types of insects undergoing incomplete and complete metamorphosis, respectively [29], [30]. Identification of candidate genes regulating wing development would provide insights into the further study about the molecular mechanisms controlling metamorphosis development. Traditionally, such gene identification in “non-model” insects relied on degenerate PCR, which is labor-intensive, expensive, prone to failure, and only produces incomplete fragments [31]. The introduction of novel high throughput sequencing technologies greatly facilitates the global analysis of the blueprint of development-related genes [32]. This technology has been used, for example, to characterize the wing development-related genes in the milkweed bug *Oncopeltus fasciatus* [33], the oriental fruit fly *Bactrocera dorsalis* [34], and the salt marsh beetle *Pogonus chalceus* [35] etc.

In this study, we combined the Illumina sequencing and *de novo* assembly to obtain and characterize the transcriptome of the wing discs of *L. migratoria* nymph. 91,907 unigenes were assembled and 23,359 ones were annotated to known databases. We also re-characterized the previous transcriptome of *O. furnacalis* larvae [36], with special emphasis on wing development-related genes. Overall, we identified 50 potential wing development-related unigenes from *L. migratoria* transcriptome, and 46 unigenes from *O. furnacalis* transcriptome, respectively. Additionally, we performed qRT-PCR analysis to investigate the gene expression profiles of several key wing development genes during the stage of rapid growth in *L. migratoria* and *O. furnacalis*. All these results provide valuable information for studying the molecular mechanisms involved in the insect wing development, and are useful resources for further exploring the mechanism how signaling pathways control wing development.

## Materials and Methods

### Insect rearing

Migratory locust *L. migratoria manilensis* was reared on fresh wheat seedlings at 28–30°C, 60% relative humidity with an 18L/6D photoperiod. Asian corn borer (*O. furnacalis* (Guenée)) larvae were reared on an artificial diet at 28°C under a relative humidity of 70–90% and a photoperiod of 18L/6D [36].

### Dissection and total RNA extraction of wing discs

The wing discs of *L. migratoria* fifth instar nymph were dissected along the wing root with small scissors under microscope. Thirty pairs of dissected wing discs were combined, and total RNA samples were prepared using TRizol Reagent (TIANGEN, Beijing, China) following the manufacturer’s instructions. Total RNA was dissolved in H_2_O and RNA quantity was determined on a Nanodrop ND-2000 spectrophotometer (NanoDrop products, Wilmington, DE, USA). RNA integrity was checked on Agilent 2100 BioAnalyzer (Agilent Technologies, Englewood, CO, USA).

### Library construction and Illumina sequencing

Ten µg of total RNA was used to isolate mRNA using oligo(dT) magnetic beads. The cDNA library was constructed using NEBNext mRNA Library Prep Reagent Set (NEB, Ipswich, MA, USA) following the manufacturer’s protocols. Briefly, enriched poly(A) RNA of each sample was fragmented into 200–700 nt pieces with RNA Fragmentation Reagents. The cleaved RNA fragments were transcribed into the first-strand cDNA using random hexamer-primers, followed by second-strand cDNA synthesis. The resulting double-stranded cDNA (dsDNA) was purified with QiaQuick PCR extraction kit (Qiagen, Hilden, Germany) and dissolved in EB buffer. The purified dsDNA was treated with T4 DNA Polymerase and T4 Polynucleotide Kinase for end-repairing and dA-tailing. After that, they were ligated to sequencing adaptors with barcode using T4 DNA ligase. Finally, fragments with around 200 bp-length were purified with QiaQuick GelPurify Kit (Qiagen, Hilden, Germany), and used as templates for PCR amplification to create the cDNA library. The library was sequenced on Illumina HiSeq 2000 (Illumina, San Diego, CA, USA) in Baimaike company (Beijing, China).

### Assembly and annotation of transcriptomes

Raw reads were filtered to remove low quality reads with Q20 less than 20 and the sequence reads containing adapters and poly-A/T tails. The resulting clean reads were assembled to produce unigenes using the short reads assembling program – Trinity [37]. For functional annotations, we first searched all unigene sequences against various protein databases such as Nr, Swiss-Prot, COG, and KEGG using BLASTX, and then searched nucleotide database Nt using BLASTN, with an E-value cut-off of 10^−5^ [38]. The BLAST results were used to extract coding region sequences (CDS) from the unigene sequences, and translate them into peptide sequences. When a unigene happened to have no BLAST hits, ESTScan software [39] would be used to determine the sequence direction. In addition, we performed the Gene Ontology (GO) annotations for each unigene with Blast2GO program according to the GO association done by a BLASTX against the Nr database [40], [41].

### Identification and sequence analysis of wing development-related genes from *L. migratoria* and *O. furnacalis* transcriptome

The available wing development-related gene sequences from *Drosophila* were used as references to screen *L. migratoria* transcriptome database obtained above and *O. furnacalis* transcriptome obtained previously [36]. The potential candidates of *L. migratoria* and *O. furnacalis* wing development-related genes were confirmed by searching the BLASTX algorithm against the non-redundant (nr) NCBI nucleotide database using a cut-off E-value of 10^−5^.

For the sequence analysis of putative wing development-related genes identified above, the deduced protein domains were determined by using Pfam (http://www.sanger.ac.uk/Software/Pfam/) and SMART (http://smart.embl.de/). Analysis of deduced amino acid sequences, including prediction of signal peptide, molecular weight and isoelectric point, was carried out in the EXPASY (Expert Protein Analysis System) proteomics server (http://www.expasy.org). Sequence comparisons and phylogenetic analysis were performed by MEGA5 software [42]. Phylogenetic trees were constructed by the neighbor-joining method, with statistical analysis by the bootstrap method, using 1000 repetitions.

### Expression assay of several identified wing development-related genes

To investigate the expression profiles of several key wing development-related genes in forewings and hind wings, we dissected the wing discs as described above, and extracted total RNA independently from 3 biological replicates. DNase I-treated RNA (1µg) was converted into first-strand cDNA using TIANScript RT Kit (TIANGEN, Beijing, China). The cDNA products were diluted 2 fold for use as template. Specific primers for each gene were designed and listed in Table S1. *L. migratoria* Actin and *O. furnacalis* ribosomal protein L8 (*rpL8*) was used as an internal standard to adjust the template amounts in a preliminary PCR experiment. The qRT-PCR was performed on a Applied Biosystems 7500 Real-time PCR system (Life Technologies, Grand Island, NY, USA) using the GoTaq qPCR Master (Promega, Madison, WI, USA), according to the manufacture’s instructions. The thermal cycling conditions for qRT-PCR and calculation methods were same as described previously [36].

## Results and Discussion

### Dissection and observation of wing discs in *L. migratoria* and *O. furnacalis*

**Figure 1:**
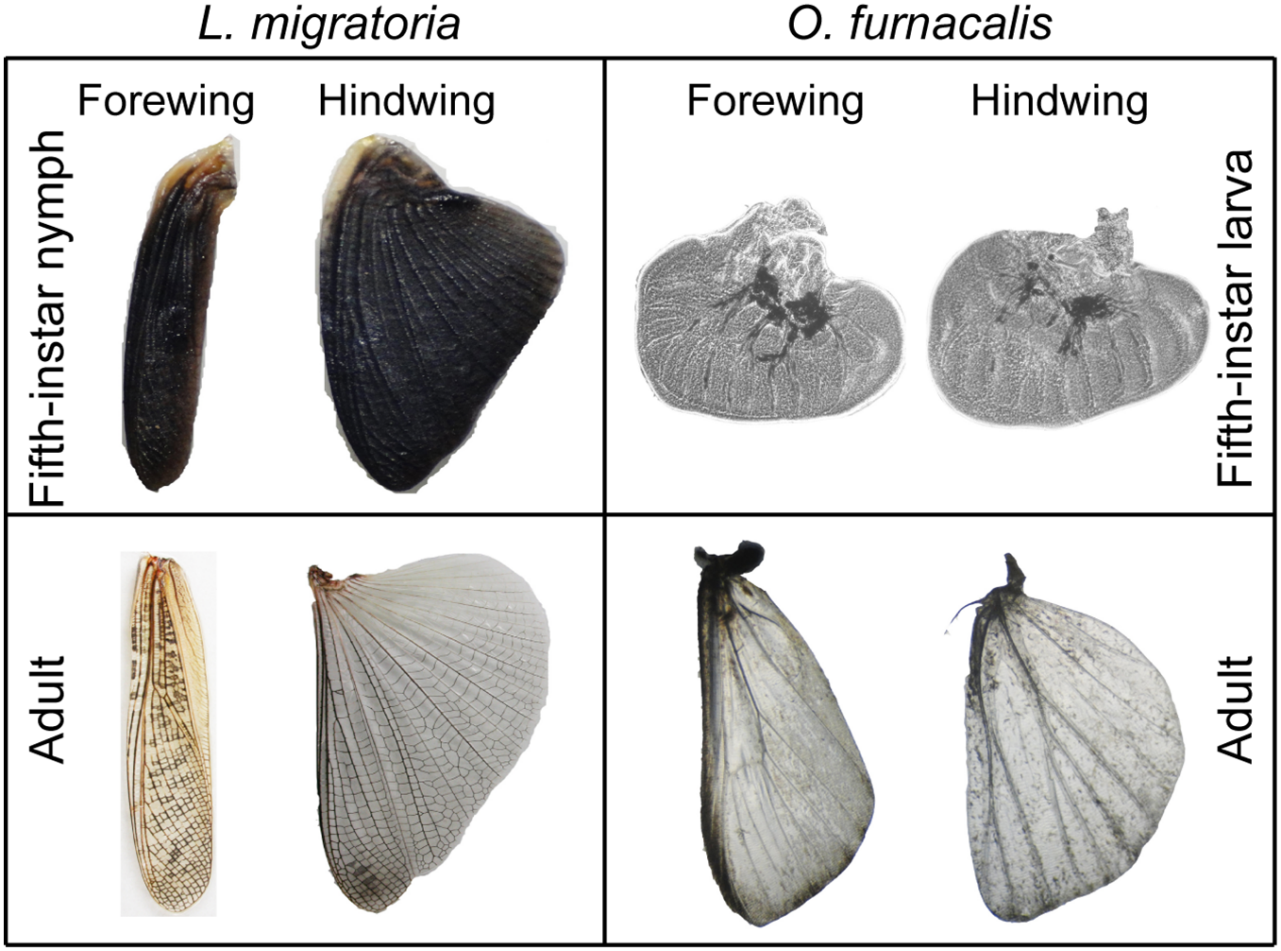
Observation and comparison of dissected wing discs and adult wings from *L. migratoria* and *O. furnacalis*. Note that *O. furnacalis* adult wings were manually removed scales.

All insects in the Pterygota undergo metamorphosis from immature to adult. Among them, insects with incomplete metamorphosis, such as migratory locust *L. migratoria*, have young nymph resembling the adult with visible forewings and hind wings. Meanwhile, insects with complete metamorphosis, such as corn borer *O. furnacalis*, go through four stage processes from egg, larva, pupa, and adult in which only adult has visible wings. Although there are some minor differences, the overall appearances of forewings and hind wings in *L. migratoria* nymph and adult are similar (Fig. 1). However, actual wings are only visible in *O. furnacalis* adult. In fifth-instar *O. furnacalis* larva, a pair of pea-like wing discs composed of large amount of cells was dissected from the position where the forewing and hind wing will be derived. There is no similarity on the appearance between the wing discs and adult wings (Fig. 1). The huge difference about the wing development in *L. migratoria* and *O. furnacalis* suggests that the molecular mechanisms controlling the wing development in insects with incomplete or complete metamorphosis might be largely different. As a first step to understand these molecular mechanisms, it is important and necessary to identify as many as possible genes functioning in the wing development. Previously, we have combined the Illumina sequencing and *de novo* assembly to obtain the high quality transcriptome from *O. furnacalis* larvae [36]. In this study, we also obtained the data for *L. migratoria* transcriptome, and we can comparatively characterize both transcriptomes and identify wing development-related genes. This work will provide useful information for studying the molecular basis involved in the wing development in *L. migratoria* and *O. furnacalis*, two insect species with different type of metamorphosis.

### Sequencing and unigene assembly of *L. migratoria* transcriptome

In order to obtain detailed information about *L. migratoria* transcriptome, we prepared cDNA from the wing discs of fifth-instar nymph, and subjected it to Hiseq 2000 sequencing. After cleaning of dirty reads and quality checks, a total of 83,000,540 high-quality clean reads (SRA accession number SRX491784) with a cumulative length of 8,382,624,748 nucleotides (8.38 Gb) were generated from *L. migratoria* wing disc library. The GC percentage of the reads is 42.65%, which is comparable with genome sequence of other insects. Using Trinity software by the manner of paired-end joining and gap-filling, these reads were assembled into 91,907 unigenes longer than 200 nt (mean length of 610 nt and N50 of 1024 nt). The size distribution indicated that the ratio of unigenes with a length of 200–1000 bp was 86.49%, while the length of 12,420 (13.51%) unigenes was more then 1000 bp (Fig.S1). It was significantly larger than that in previous insect transcriptome projects [43], [44]. The assembled sequences have been deposited in the NCBI Transcriptome Shotgun Assembly (TSA) Database under the accession GBDZ00000000.

### Functional annotation and classification

In order to annotate the unigenes, the obtained sequences were first aligned by BLASTX to various protein databases of nr, Swiss-Prot, KEGG, COG, and GO (E-value<10^−5^), and then aligned by BLASTN to nucleotide database nt (E-value<10^−5^). Among 91,907 unigenes, 20,746 (22.6%), 8,737 (9.5%), 11,450 (12.5%), 5,312 (5.8%), 4,986 (5.4%), and 10,644 (11.6%) ones were annotated in nr, nt, Swiss-Prot, KEGG, COG, and GO, respectively (Table S2). A total of 23,359 unigenes (25.4%) were annotated to at least one database. The remaining 68,548 unigenes (74.6%) were not annotated to any referred databases. The low annotated percentage might be due to the transcripts derived from the cDNA of untranslated regions, assemblage errors, and nonconserved areas of proteins where homology is not detected [43]. Another possibility was that a large part of the genes in *L. migratoria* transcriptome database were with unknown functions, and the un-annotated unigenes were the potential sources of novel genes.

**Figure 2:**
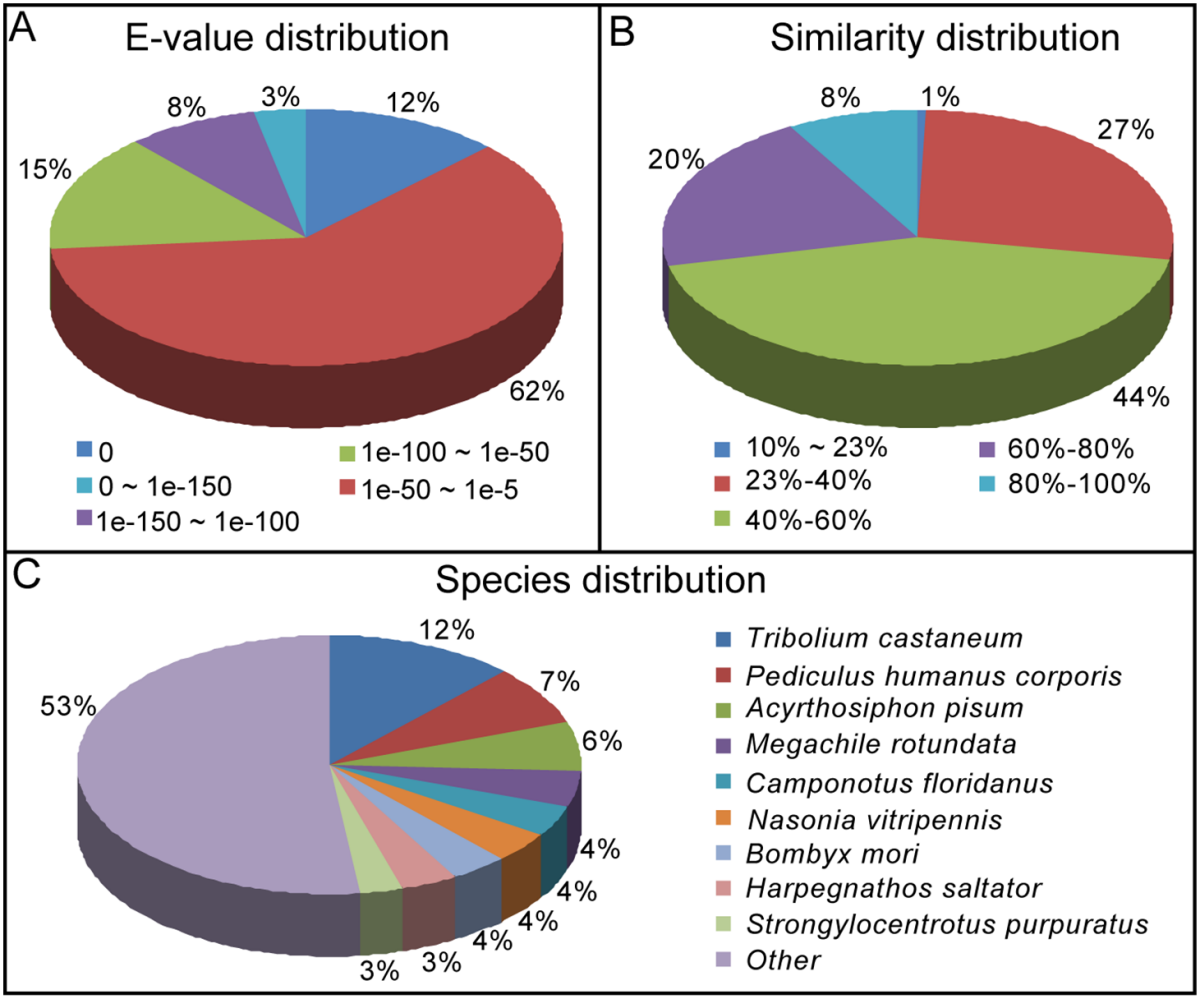
Homology analysis of *L. migratoria* unigenes. (A) E-value distribution. (B) Similarity distribution. (C) Species distribution. All unigenes that had BLASTX annotations within the NCBI Nr database with a cut-off E-value of 10–5 were analyzed. The first hit of each sequence was used for analysis.

The functional annotations of unigenes were performed mainly based on the BLASTX results against the nr database. Among the 20,746 unigenes annotated to nr database, 7,883 (38%) showed strong homology (E-value smaller than 1e-50) (Fig. 2A). The identity comparison showed 5,809 (28%) unigenes have more than 60% identity with other insects (Fig. 2B). For species distribution of the top BLAST hits against the Nr databse, *L. migratoria* unigenes had the greatest number of matches with *Tribolium castaneum* (2,490 unigenes, 12%), followed by *Pediculus humanus corporis* (1,452 unigenes, 7%), and *Acyrthosiphon pisum* (1,245 unigenes, 6%) (Fig. 2C).

**Figure 3:**
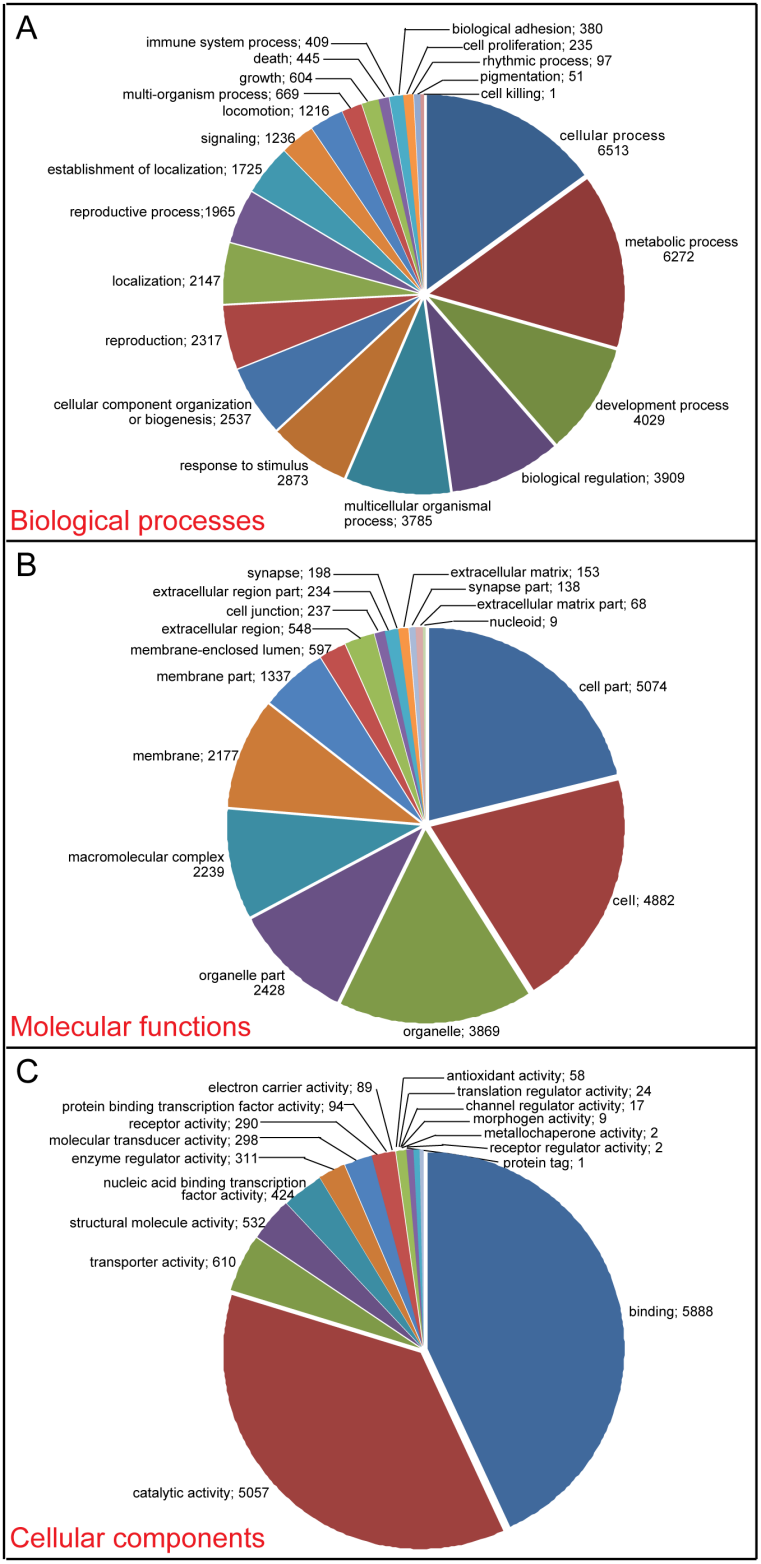
Gene ontology (GO) assignment for the *L. migratoria* transcriptome. GO assignments (level 2) as predicted for their involvement in (A) biological processes, (B) cellular components, and (C) molecular functions. The number of unigenes assigned to each GO term is shown behind semicolon.

Gene Ontology (GO) assignment programs were used to classify the functions of all unigenes. GO contains three categories: biological process, cellular component, and molecular function. Using the Blast2GO and WEGO software, 43,415, 24,188, and 13,706 unigenes were associated with biological process (22 sub-categories), cellular component (16 sub-categories), and molecular function (17 sub-categories), respectively (Fig. 3). Among the biological process assignments, cellular processes (15.0%) and metabolic processes (14.4%) represented the most abundant subcategories. It indicated the importance of cell cycle, generation as well as metabolic activities in wing development stage. Under the category of cellular component, the top 3 sub-categories were cell part (21.0%), cell (20.2%) and organelle (16.0%). In the molecular function category, binding (43.0%) and catalytic activities (36.9%) were the most abundant (Fig. 3). The subcategories taking up the largest two proportions in each category were consistent with that in transcriptomic studies of other insects [45], [46].

**Figure 4:**
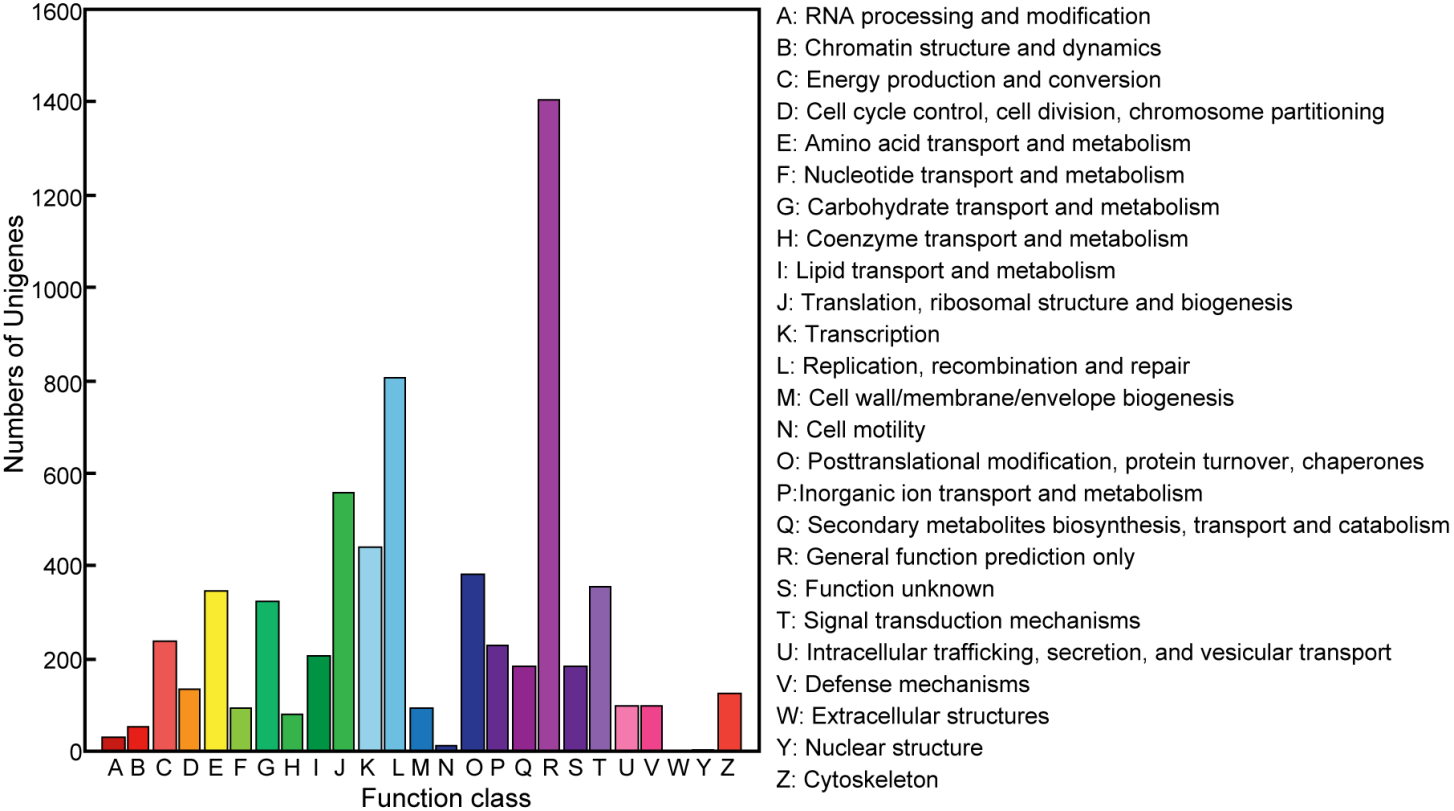
Clusters of orthologous groups (COG) classification of *L. migratoria* unigenes. A total of 6,508 produced functional annotations were among the 25 categories. The Y-axis shows the number of unigene in each COG term.

The unigenes were further compared against COG for the analysis of the putative protein functions. A total of 6,508 unigenes were functionally classified into 25 COG categories (Fig. 4). The cluster for ‘General function prediction only’ (1,407 unigenes) constituted the largest group, followed by ‘Replication, recombination and repair’ (809 unigenes), ‘Translation, ribosomal structure and biogenesis’ (560 unigenes), and ‘Transcription’ (441 unigenes) (Fig. 4). The group of ‘signal transduction mechanisms’ contains 354 unigenes, the fifth largest one within the COG classification of the *L. migratoria* transcriptome. It suggests the possible importance of signaling transduction pathways in wing development in *L. migratoria*.

### Identification and comparison of wing development-related genes in *L. migratoria* and *O. furnacalis* transcriptome

Wing development is a complicated biological process comprising multiple integrated signaling pathways. In particular, four signaling pathways – Notch, Hedgehog (Hh), Decapentaplegic (Dpp), Wingless (Wg) – play important roles in growth control and cell fate determination during the wing development [20], [22], [25], [47]–[49]. In order to obtain the global perspective on the wing development-related transcripts in *L. migratoria*, we searched the assembled transcriptome for orthologous genes known to be involved in the above four pathways in *Drosophila*. In total, we have identified 50 unigenes with significant similarity to *Drosophila* wing development-related genes, including 7 ones in Notch pathway, 12 in Hh pathway, 9 in Dpp pathway, and 10 in Wg pathway (Table 1). Meanwhile we performed the similar analysis for *O. furnacalis* transcriptome reported previously [36], and identified 46 unigenes with high potential to be related to the wing development (Table 1).

**Table 1:** Summary of the wing development-related unigenes identified in *L. migratoria* and *O. furnacalis* transcriptome.

		*Locusta migratoria*							*Ostrinia furnacalis*						
	Function^a^	UnigeneID	NucleotideLength(nt)	Fulllength^b^		Proteinlength (aa)^c^	Identity to Besthit (%)^d^	P value^e^	Unigene ID	Nucleotidelength(nt)	Fulllength^b^		ProteinLength(aa)^c^	Identity to Besthit (%)^d^	P value^e^
**Hedgehog pathway**															
hedgehog (hh)	L	a37032	4051	3′		383	66	e-101	Unigene16362	590	M		196	73	9e-064
patched (ptc)	R	a57898	1327	5′		372	75	e-135	Unigene153	3111	Y		775	62	0.0
smoothened (smo)	R	a59513	2125	3′		651	62	6e-073	CL2764.Contig1	2937	Y		751	67	0.0
cubitus interruptus (ci)	F	a69651	650	M		216	79	4e-096	Unigene15615	910	M		303	77	5e-098
costal-2 (co-2)	B	a8431	3970	Y		843	52	e-102	?						
Fused	K	a32509	2482	3′		650	74	7e-071	Unigene11591	1346	M		448	64	1e-071
suppressor of fused (sufu)	B	a11681	2472	3′		488	55	3e-086	Unigene16575	637	M		212	59	3e-042
**Decapentaplegic pathway**															
decapentaplegic (dpp)	L	a136595	815	3′		209	69	1e-068	Unigene7326	990	3′		306	63	1e-078
glass bottom boat (gbb)	L	a2659	1653	3′		332	64	5e-077	CL9574.Contig1	1573	Y		448	61	8e-097
saxophone (sax)	R	a14241	2261	Y		568	76	0.0	Unigene21017	796	M		256	77	1e-098
punt	R	a17842	5910	Y		503	69	e-144	CL628.Contig1	2051	Y		547	49	1e-051
thickveins (tkv)	R	a4697	2436	Y		512	72	e-174	Unigene5852	2162	3′		335	82	e-137
mothers against Dpp (mad)	F	a11173	2860	Y		475	84	0.0	Unigene16360	1775	Y		423	86	0.0
		a12619	3021	Y		436	59	e-152	Unigene6852	1575	Y		447	58	e-147
medea (med)	A	a26380	1235	3′		356	67	1e-095	Unigene4133	440	M		146	89	3e-045
		a11651	3401	M		397	66	e-131	Unigene17363	589	M		196	89	3e-080
optomotor-blind (omb)	F	a4984	803	M		267	96	e-121	CL105.Contig5	1131	M		376	89	9e-142
spalt major (salm)	F	a6922	1073	5′		325	55	2e-070	CL2350.Contig2	2278	M		759	54	2e-097
spalt related (salr)	F	a12971	2087	5′		651	47	2e-089	?						
**Notch signaling Pathway**															
Notch (n)	R	a1269	12296	Y	2484		72	0.0	Unigene6905	2340	M	780		83	0.0
delta (dl)	L	a123129	926	M	308		69	e-114	Unigene3068	987	M	328		69	e-120
serrate (ser)	L	a1910	7128	Y	1324		49	0.0	CL7020.Contig1	1927	M	409		52	e-164
Hairless (h)	R	a35245	1975	5′	538		61	5e-032	Unigene3346	243	M	80		68	4e-015
suppressor of hairless (su(H))	A	a4077	3351	Y	496		95	0.0	Unigene16575	637	M	211		59	3e-042
mastermind (mam)	A	a14121	1140	5′	153		54	1e-025	Unigene19131	1464	5′	487		54	3e-036
notchless	B	a6959	1804	Y	474		78	0.0	CL2896.Contig1	1054	5′	281		80	e-116
fringe	K	a17301	2466	3′	259		82	e-111	Unigene9365	1276	Y	327		73	e-100
hairy	B	a4291	3634	3′	407		52	6e-070	Unigene22517	673	5′	100		72	3e-32
**Wingless pathway**															
wingless (wg)	L	a40139	948	3′	227		85	7e-050	Unigene1219	292	M	97		65	4e-020
frizzled (fz)	R	a1577	5642	Y	571		68	0.0	CL4000.Contig1	1705	Y	497		71	e-157
frizzled2 (fz2)	R	?							Unigene2716	1952	5′	516		57	e-113
arrow (arr)	R	a39913	4189	5′	1381		65	0.0	Unigene11432	3712	5′	1167		67	0.0
disheveled (dsh)	F	a6911	3503	Y	721		60	e-154	Unigene24227	256	M	85		100	3e-044
armadillo (arm)	F	a1041	3398	Y	820		87	0.0	Unigene21327	2918	Y	737		84	0.0
axin	A	a4962	3922	3′	676		64	2e-031	Unigene17645	493	M	164		53	8e-023
adenomatous polyposis coli (APC)	A	a20732	4723	5′	1541		47	e-160	Unigene11483	2788	5′	880		60	e-156
vestigial (vg)	F	a302963	243	M	81		62	4e-013	Unigene4539	380	M	126		55	2e-021
scalloped (sd)	F	a1575	4661	Y	459		69	0.0	CL1169.Contig4	2469	Y	413		61	2e-167
shaggy	K	a2857	10994	Y	1913		83	0.0	Unigene16270	3819	Y	428		86	e-176
**Others**															
ultrabithorax (Ubx)	F	a12834	2418	3′	174		56	6e-053	Unigene20941	1207	Y	253		46	6e-068
apterous (ap)	F	a188016	391	M	130		59	5e-044	Unigene4961	1440	3′	348		53	1e-098
engrailed (en)	F	a1647	2530	3′	138		80	3e-052	CL5152.Contig1	2062	Y	517		70	5e-057
homothorax (Hth)	F	a22527	721	M	240		75	e-102	CL5887.Contig2	1800	3′	235		77	2e-099
teashirt (Tsh)	F	a866117	347	M	115		55	8e-027	?						
epidermal growth factor receptor (EGFR)	R	a1492	4431	Y	1394		53	0.0	CL625.Contig2	6301	Y	1458		60	0.0
rhomboid (rho)	K	a36342	2527	Y	294		58	1e-072	?						
nubbin (Nub)	F	a3913	1608	3′	460		45	1e-080	Unigene16843	2180	Y	355		65	2e-052
pannier (Pnr)	F	a455482	478	M	159		71	7e-053	Unigene16014l	2513	3′	341		46	3e-065
notum	K	a538614	267	M	88		62	9e-029	Unigene26847	643	M	214		47	1e-046
fat (ft)	R	a9170	5093	M	1697		51	0.0	Unigene18347	1879	M	626		55	0.0
four-jointed (fj)	K	a37684	381	M	127		66	9e-036	Unigene6909	1600	3′	530		41	e-107
dally-like (dlp)	B	a6329	2624	Y	629		51	e-104	?						

a The mark of L, R, A, F, K, and B means that the unigens potentially function as ligand, receptor, transcription activator, transcription factor, protein kinase, or binding protein.

b The mark of Y, 5′, 3′, and M means that the fragment of the unigene consists of complete open reading frame, 5′-end containing start codon, 3′-end containing stop codon, and the middle part without start and stop codons, respectively.

c The number indicates the length of amino acid sequences deduced from available coding region, which is complete or not.

d Identity indicates the percentage of identical amino acid residues in unigenes to the sequence best hit in BLASTX.

e P-value is determined by FDR (false discovery rate).

#### Genes involved in the Hh signaling pathway

**Figure 5:**
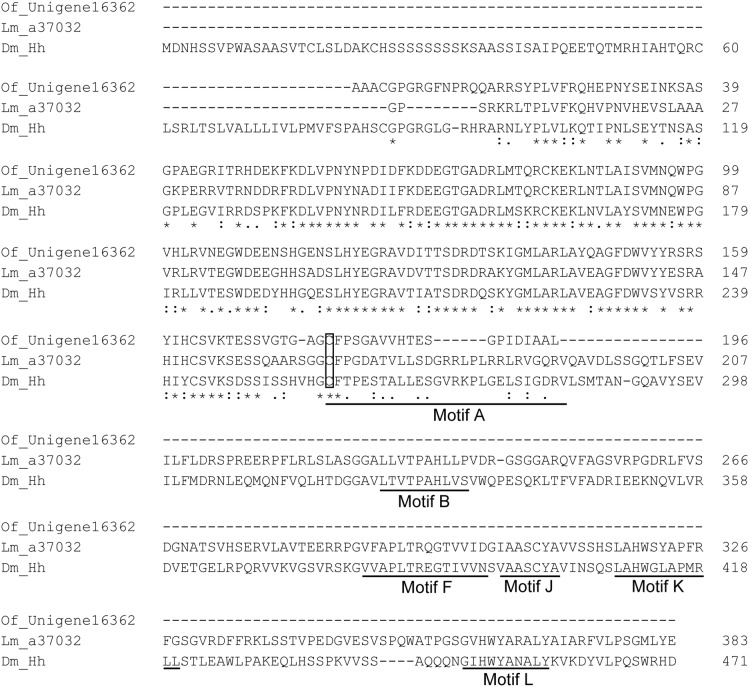
Alignments of potential Hhs in *L. migratoria*, *O. furnacalis* and *Drosophila*. The deduced amino acid sequences of *L. migratoria* a37032 and *O. furnacalis* Unigene16362 were compared with *Drosophila* Hh (Dm_Hh). Completely conserved amino acids are indicated by “*”, and conservative substitutions by “:” and “.” below the sequences. The conserved cysteine residue for an acyl rearrangement analogous to step 1 of the protein splicing pathway is boxed. The conserved motifs in *Drosophila* Hedgehog are underlined.

**Figure 6:**
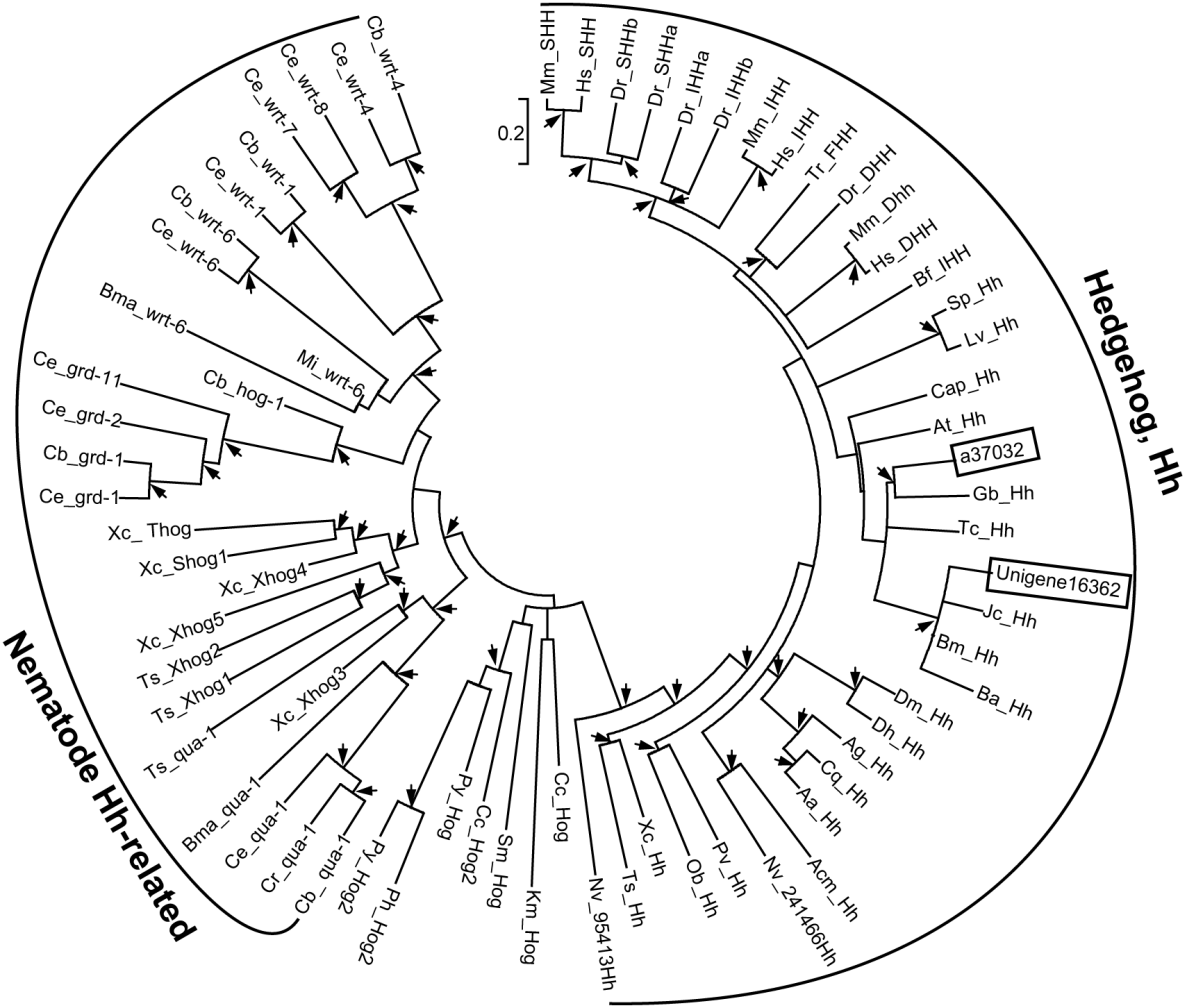
Phylogenetic analysis of Hhs. The amino acid sequences of *L. migratoria* a37032 and *O. furnacalis* Unigene16362, together with 68 potential Hhs from other organisms were used to build the neighbor joining tree. *L. migratoria* a37032 and *O. furnacalis* Unigene16362 are boxed. Nematode Hh-related proteins and other eukaryote Hh proteins are indicted with brackets. The arrows at nodes denote bootstrap value greater than 700 from 1000 trials.

**Figure 7:**
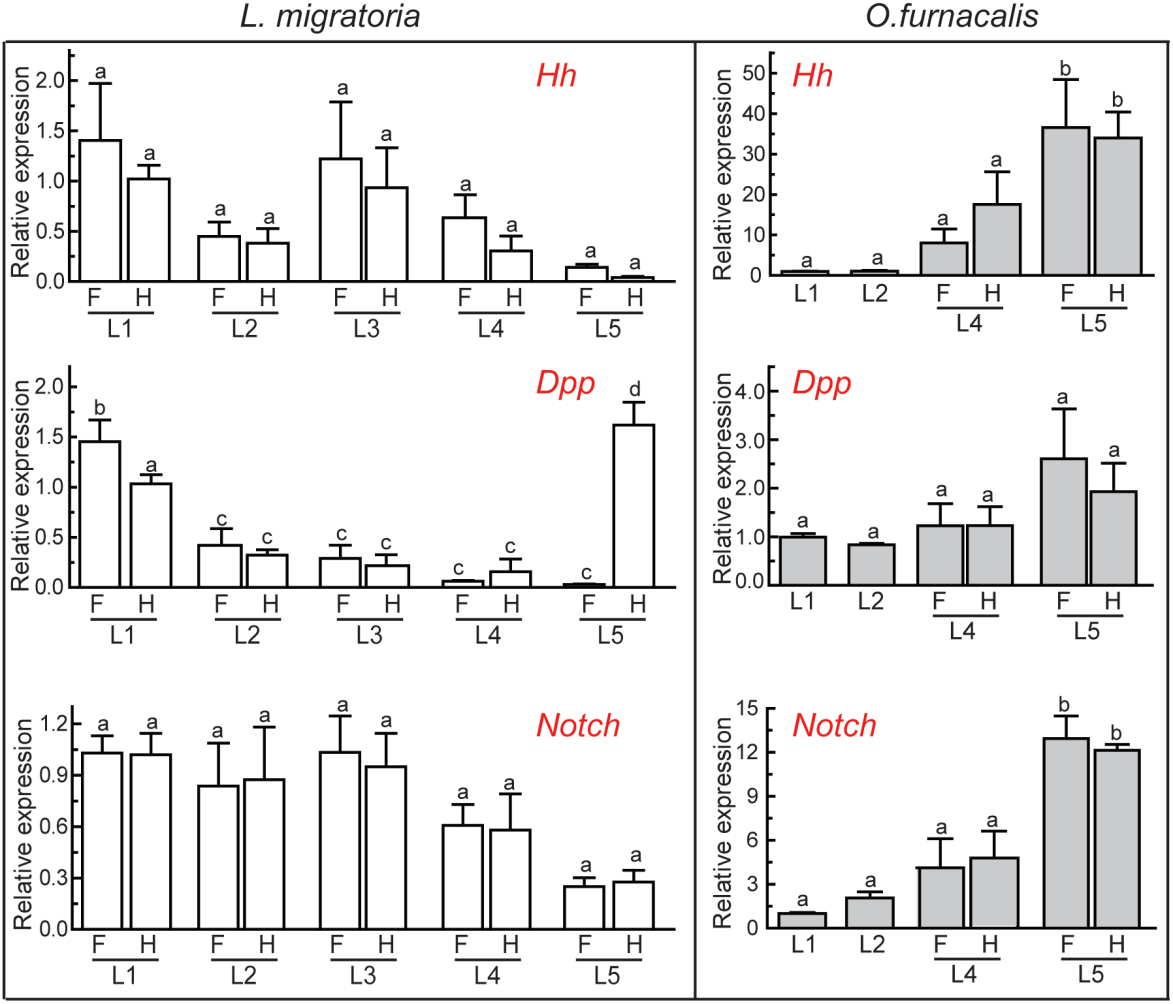
qRT-PCR analysis of transcriptional levels of *hh*, *dpp*, and *Notch* in *L. migratoria* and *O. furnacalis*. *Actin* and ribosomal protein L8 (*rpL8*) were used as an internal standard to normalize the templates in *L. migratoria* and *O. furnacalis*, respectively. RNA was extracted from wing discs of forewings (F) or hind wings (H) collected from the first-instar (L1), second-instar (L2), third-instar (L3), fourth-instar (L4) and fifth-instar (L5) *L. migratoria* nymph or *O. furnacalis* larvae. The bars represent the mean ± S. D. (*n* = 3). Bars labeled with different letters are significantly different (one-way ANOVA, followed by the Newman-Keuls test, *P*<0.05).

The Hh pathway is an important signaling cascade in *Drosophila* to pattern the embryonic cuticles and adult appendages [50]. It is also vital for diverse aspects of animal development and essential in humans to pattern their limbs and internal organs [51], [52]. The pathway takes its name from its polypeptide ligand, an intercellular signaling molecule called as Hh [53]. *Hh* was initially discovered as a segment polarity gene in *Drosophila* [54]. Mammals have three Hh orthologues, Sonic (Shh), Indian (Ihh), and Desert (Dhh) [55]. We have identified the potential *hh* genes, a37032 from *L. migratoria* and Unigene16362 from *O. furnacalis* transcriptome, respectively (Table 1). Although these two unigenes were incomplete, they have 66% and 73% amino acid sequence identity to *Drosophila* Hh (Fig. 5). On the other hand, *Drosophila* Hh is synthesized as inactive precursors with an N-terminal signaling region linked to a C-terminal autoprocessing region which begins with a cysteine for an acyl rearrangement analogous to step 1 of the protein splicing pathway [56]. This Cys residue is completely conserved in both *L. migratoria* and *O. furnacalis* Hh homologues (Fig. 5). The conserved motif A, B, F, J, K, and L in *Drosophila* Hh [57] were found in *L. migratoria*, but not in *O. furnacalis* Hh because its C-terminus was presently unknown (Fig. 5). The phylogenetic analysis of Hh from different insect species and other animals reveals that almost all nematode Hh-related proteins form a distinct clade while the other eukaryote Hh proteins, including *L. migratoria* a37032 and *O. furnacalis* Unigene16362, cluster into another Hh clade (Fig. 6). *L. migratoria* a37032 is grouped with other Orthopteran Hhs, and *O. furnacalis* Unigene16362 is clustered with other Lepidopteran Hhs. We performed qRT-PCR analysis to analyze the transcriptional expression of *hh* during different developmental stages. The mRNA abundance of *hh* in *L. migratoria* remained unchanged from the first instar through the fifth instar stage. The transcript abundance of *O. furnacalis hh* was consistent before the fourth instar stage, but increased significantly in the fifth instar larvae (Fig. 7). This result also suggests that the quality of the transcriptome assembly is high enough to be used for primer design.

Hh signaling is mediated by a multi-component receptor complex in the cell membrane. This receptor complex consists of a 12-span transmembrane protein, Patched (Ptc) as the receptor and a 7-span transmembrane protein, Smoothened (Smo) as the obligatory signal transducer across the plasma membrane [51], [58]. When extracellular Hh binds to and is inhibited by Ptc, Smo starts to accumulate, and inhibit the proteolytic cleavage of zinc-finger transcription factor Cubitus interruptus (Ci) which is normally bound by the kinesin-like protein Costal-2 (Cos2). The intact Ci protein then translocate into the nucleus, allowing the transcription of some genes such as *dpp*. In the absence of Hh, Ptc blocks Smo activity, and full-length Ci protein is degraded into Ci fragment (CiR) that functions as a transcriptional repressor to block the transcription of target genes (Fig. S3A) [50]. By searching *L. migratoria* and *O. furnacalis* transcriptome using *Drosophila* corresponding genes as reference, we have identified the potential 1∶1 orthologs of Ptc, Smo, Ci from both transcriptome databases (Table 1). It suggests that Hh signaling pathway is conserved in both *L. migratoria* and *O. furnacalis*.

#### Genes involved in Dpp signaling pathway

Dpp, directed by the Hh signaling, is a member of the bone morphogenetic protein (BMP) growth factor family [59]. Dpp forms a long-range dynamic and precise gradients to control cell survival, cell morphogenesis, cell proliferation, cell differentiation during the wing development [22], [25], [26], [60]. When a cell receives a Dpp signal, its heteromeric receptor complex composed of type I receptor Thickveins (Tkv) and type II receptor Punt is activated [61], [62]. Once activated, the receptors are able to phosphorylate an intracellular protein called mothers against Dpp (Mad) [63]. The phosphorylated Mad then associate with the Medea (Med), and the complex translocates to the nucleus where it binds to DNA and activates or suppresses the expression of the target genes in conjunction with other transcription factors [64]. Within this signaling pathway, the *Drosophila* Hox gene *Ultrabithorax* (*Ubx*) restricts both the transcription and the mobility of Dpp [65]. Genes activated by Dpp signaling pathway include transcription factor *optomotor-blind* (*omb*) and two members of Spalt (sal) family *spalt major* (*salm*), *spalt related* (*salr*) and so on (Fig. S3B) [66], [67].

**Figure 8:**
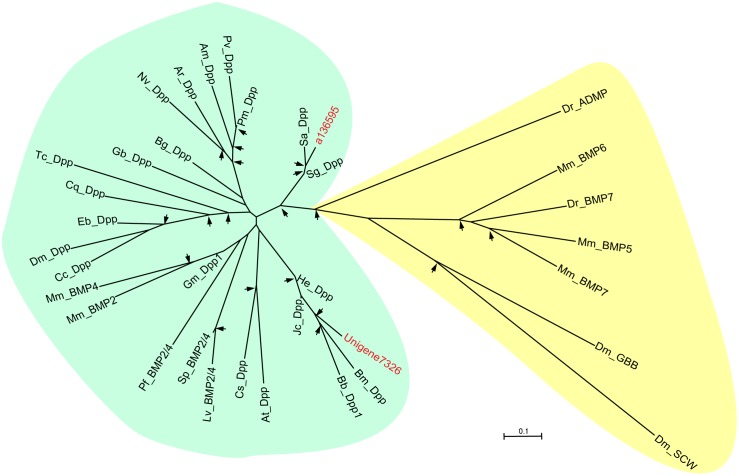
Phylogenetic analysis of Dpps. Except for *L. migratoria* a136595 and *O. furnacalis* Unigene7326 (marked in red), the used amino acid sequences of other 33 Dpps are from mouse (Mm), zebrafish (Dr), lancet (*Branchiostoma floridae*; Bf), acorn worm (*Ptychodera flava*; Pf), the sea urchins *Strongylocentrotus purpuratus* (Sp) and *Lytechinus variegatus* (Lv), fruit fly (Dm), flour beetle (*Tribolium castaneum*; Tc), sawfly (*Athalia rosae*; Ar), buckeye butterfly (*Junonia coenia*; Jc), the grasshoppers *Schistocerca americana* (Sa) and *S. gregaria* (Sg), cricket (*Gryllus bimaculatus*; Gb), *Cupiennius salei* (Cs), *Ceratitis capitata* (Cc), *Blattella germanica* (Bg), *Polyrhachis vicina* (Pv), *Episyrphus balteatus* (Eb), *Apis mellifera* (Am), *Pheidole morrisi* (Pm), *Biston betularia* (Bb), *Bombyx mori* (Bm), *Culex quinquefasciatus* (Cq), *Nasonia vitripennis* (Nv). The branches specific for arthropod Dpps and TGF-beta homologs are shaded in blur and yellow, respectively. For explanation of the arrows see Fig. 6.

In this study, we identified almost all transcripts of the above key components in Dpp signaling pathway (Table 1). Among them, one unigene with sequence similarity to *Drosophila* Dpp was identified from the transcriptome of *L. migratoria* (a136595) and *O. furnacalis* (Unigene7326), respectively. The 5′-end of the cDNA sequences of both potential *Dpp* is incomplete. In order to establish the orthology of these two fragments, we performed a phylogenetic analysis incorporating a selection of BMP growth factor members from *Drosophila*, other arthropods and a variety of deuterostome taxa. The resulting phylogenetic tree distinguishes two groups of proteins. One group consists of the arthropod *dpp* genes and their deuterostome homologs, the BMP2/4 genes. The second group comprises the *Drosophila* TGF-beta genes *screw* (*scw*) and *glass bottom boat* (*gbb*), and the remaining deuterostome BMP genes with TGF-beta homology (Fig. 8). The identified fragments, *L. migratoria* a136595 and *O. furnacalis* Unigene7326, reside in the *dpp* group with statistical support (reliability value = 98 and 99 for a136595 and Unigene7326, respectively). Therefore, they are postulated as potential *dpp* genes. We conducted qRT-PCR analysis to investigate the expression profile of *dpp*. *L. migratoria dpp* was expressed at high level in the first instar nymphs, then decreased significantly in the second, third, and fourth instar nymphs, but significantly increased in the hind wings of the fifth instar nymph. The mRNA level of *O. furnacalis dpp* remained unchanged in all tested stages (Fig. 7). The different expression patterns of *dpp* in two insects suggest that *dpp* might be related to the different metamorphosis.

In addition, it is interesting that only one *mad* gene was present in *Drosophila* but two transcripts encoding for potential *mad* were identified from both *L. migratoria* and *O. furnacalis* transcriptomes (Table 1). Two Mads in *L. migratoria* (a11173 and a12619) shared 60% identity while two *O. furnacalis* Mads (Unigene16360 and Unigene6852) had 61% identity in amino acid sequences. A similar situation happened to the identification of *med*. There is only one *med* gene in *Drosophila* but two transcript fragments were identified for both *L. migratoria* and *O. furnacalis med*, including a26380 and a11651 in *L. migratoria*, and unigene4133 and unigene17363 in *O. furnacalis*, respectively. The encoded amino acid sequences of a26380 and unigene4133 were highly similar to the N-terminus of *Drosophila* Med while a11651 and unigene17363 were highly similar to the C-terminus of *Drosophila* Med (Fig. S2). We doubted that the two unigenes from each transcriptome were just two partial fragments within *med* gene. However, no overlapped sequences were observed in a26380 and a11651, or in unigene4133 and unigene17363 (Fig. S2). One possible reason might be due to the sequencing errors because the predicted overlapping part was just located at the terminus of sequenced fragment. Further experiments are required to determine the full length of *med* in these two insect species.

#### Genes involved in the Notch signaling pathway

**Figure 9:**
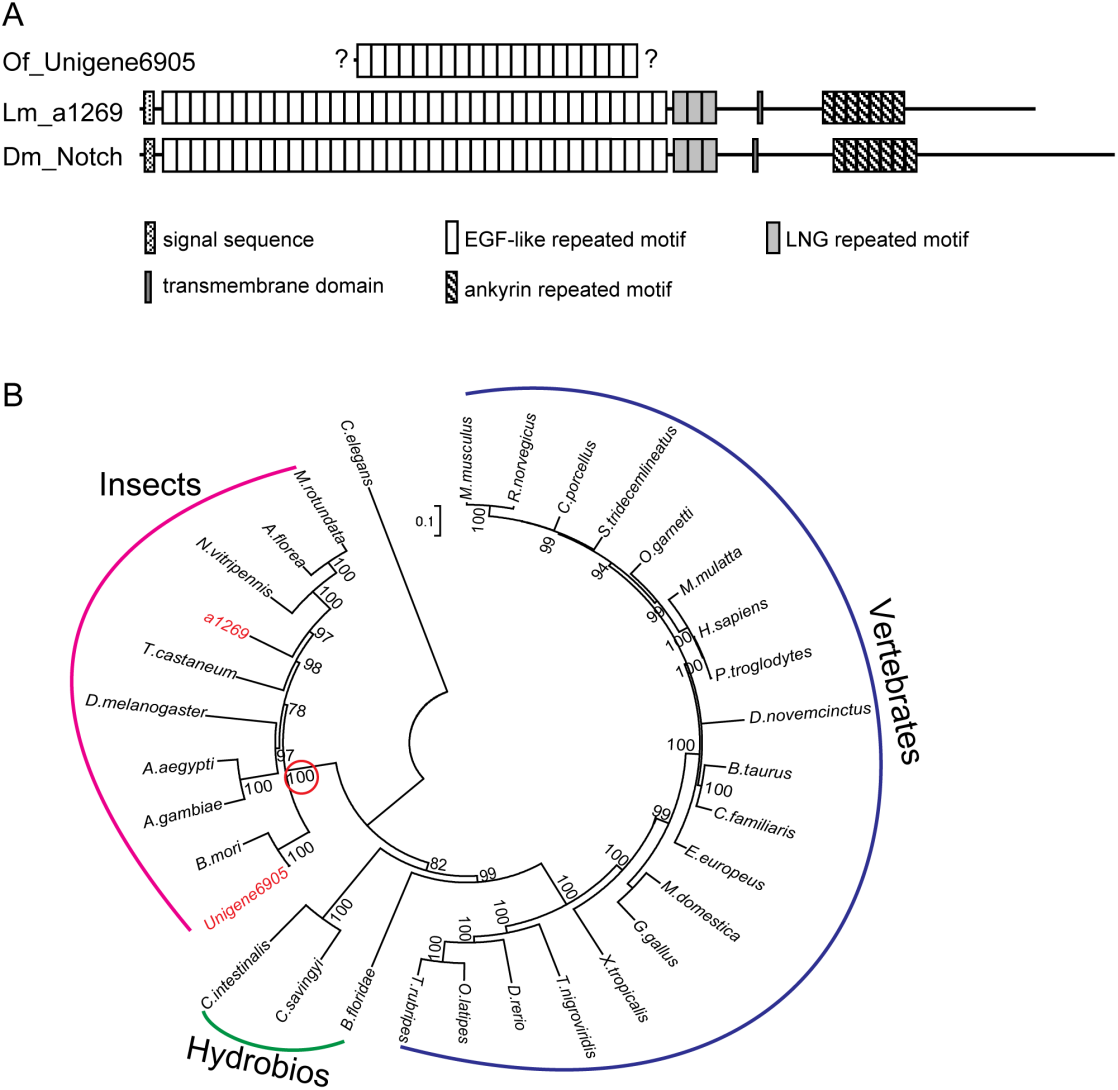
(A) Schematic representation of the *L. migratoria* and *O. furnacalis* Notch. The domain organization was predicted using the SMART program (http://smart.embl.de/). Question mark means the end is incomplete. (B) Phylogenetic analysis of the Notch from insects, hydrobios and vertebrates. The unrooted tree was generated using the distance-based method Neighbor-Joing, with *Caenorhabditis elegans* protein as outgroup. The branch lengths reflect evolutionary divergence. The circled bootstrap value indicates that *L. migratoria* a1269 and *O. furnacalis* Unigene6905 (marked in red) belong to insect Notch group.

The Notch signaling pathway is highly conserved throughout the animal kingdom. It regulates cell-fate determination during development and maintains adult tissue homeostasis [47]. The key component of this signaling pathway is the Notch receptor. Notch is a 300 kDa single-pass transmembrane protein, composed of a large extracellular domain, a single transmembrane portion, and a small intracellular region [68]–[70]. After binding to its ligands, Delta and Serrate (known as Jagged in mammals), inactive Notch precursor undergoes two proteolytic cleavage events: the first cleavage is catalyzed by ADAM-family metalloproteases; the second cleavage is mediated by γ-secretase which is an enzyme complex containing presenilin, nicastrin, PEN2 and APH1 [71]. The second cleavage liberates the Notch intracellular domain (NICD), which then migrates into the nucleus and cooperates with the DNA-binding protein CSL (also named as CBF1, Suppressor of hairless (Su(H)), LAG-1, or RBP) and its co-activator Mastermind (Mam) to promote the transcription of downstream target genes, such as *wg* (Fig. S3C) [72]. Using the corresponding components in *Drosophila* as referred sequences, we have identified 9 transcripts for potential components in Notch signaling pathway from *L. migratoria* and *O. furnacalis* transcriptomes, respectively (Table 1). Four out of nine unigenes in *L. migratoria* are complete while only one unigene is complete in *O. furnacalis*. The possible reason was that the sequencing quality of *L. migratoria* transcription was better than that of *O. furnacalis* transcriptome (8.38 Gb vs. 4.72 Gb). Given the importance of Notch in this signaling pathway, we selected it for further analysis. Similar to the case in other invertebrates, we only identified one transcript for Notch gene, a1269 from *L. migratoria* transcriptome, and Unigene6905 from *O. furnacalis* transcriptome (Table 1). The canonical Notch protein consists of three repeated sequence motifs: the extracellular domains contain 10∼36 copies of an ∼40-amino acid epidermal growth factor-like (EGFL) sequence motif and 3 copies of an ∼40-amino acid *lin*/*Notch*/*glp* (LNG) sequence motif; the intracellular domains contain 6–7 copies of a *cdc10*/*SW16*/ankyrin (CDC/ANK) sequence motif flanked by stretches of nonrepetitive sequences [73]. *L. migratoria* a1269 encodes a 2,484-amino acids full-length protein which has 63% similarity to *Drosophila* Notch. *L. migratoria* Notch contains 36 EGFL tandem repeats, 3 LNG repeats, and 7 tandem ANK repeats (Fig. 9A), suggesting it is a canonical Notch. *O. furnacalis* Unigene6905 encodes a 780-amino acids polypeptide with 70% similarity to *Drosophila* Notch. Only 20 tandem EGFL repeats are predicted in the current identified Unigene6905 fragment (Fig. 9A). It is unknown whether *O. furnacalis* Notch also contain classic LNG and ankyrin repeats because the current transcript is incomplete. We performed the phylogenetic analysis for *L. migratoria* and *O. furnacalis* Notch and 30 Notch sequences from other species to investigate the evolutionary relationship of the Notch protein family. As shown in Fig. 9B, the Notch from insects forms a separate group which includes *L. migratoria* and *O. furnacalis* Notch, and the Notch from vertebrates is clustered into another group. Notch from *Ciona*, sea urchin, and amphioxus is grouped with vertebrate Notch, however, with a low bootstrap value of 82. *Caenorhabditis elegans* Notch is out of any group, showing a great differentiation from the other taxa. We analyzed the expression profiles of *Notch* using qRT-PCR methods. As shown in Fig. 7, the expression profile of *Notch* was similar to that of *Hh* in two insects. It kept unchanged from the first instar through the fifth instar stage in *L. migratoria*, while it increased significantly in *O. furnacalis* fifth instar larvae (Fig. 7).

#### Genes involved in the wg signaling pathway

Wg (the vertebrate homolog of which is Wnt) is transcriptionally activated by Notch signaling, and Wg signaling pathway plays critical roles in axis patterning, cell fate specification, cell proliferation, and cell migration etc [74]. During the development of *Drosophila* wing, canonical Wg signaling pathway specifies pattern formation along the dorsal/ventral (D/V) axis while Dpp signaling pathway play this role along the A/P axis [75]. The ligand Wg is the founding member of the Wnt family, and is a secreted lipid-modified signaling glycoprotein that has 350–400 amino acids in length [76]. Wg is expressed at the D/V boundary and forms a stable and long-range gradient by symmetrically diffusing at both sides of the boundary. When Wg binds to a receptor complex consisting of the seven-transmembrane protein Frizzled (Fz) and the single-pass transmembrane protein Arrow (Arr, homologous to murine and human low density lipoprotein (LDL) receptor-related protein 5 or 6 (LRP5/6)), the downstream cytoplasmic protein disheveled (Dsh in *Drosophila* and Dvl in vertebrates) is activated [77]. Dsh in turn inhibits glycogen synthase kinase (GSK)-3β in the β-catenin destruction complex, which mainly consists of Axin, GSK-3β, adenomatous polyposis coli (APC) and β-catenin (armadillo (arm) in *Drosophila*). Consequently, β-catenin accumulates in the cytoplasm, and stabilized β-catenin then translocates into the nucleus and acts together with the transcription factor Pangolin to regulate the transcription of Wg target genes (Fig. S3D) [77]. In this study, we identified 10 and 11 unigenes for the known components in the Wg signaling pathway from *L. migratoria* and *O. furnacalis* transcriptome, respectively (Table 1). No putative *Frizzled 2* (*fz2*) ortholog was identified in *L. migratoria* transcriptome. A possible reason is that *fz2* gene is missing in *L. migratoria* because of the evolutionary event. The other reason with higher possibility is that the transcript level of *fz2* is low and it is not captured in the RNA-seq. We attempted to perform further analysis for identified *wg* gene, a40139 in *L. migratoria* and Unigene1219 in *O. furnacalis*. However, unigene1219 only encodes a 97-amino acid peptide which is too short to have no common sequences with other Wg during the alignment. Therefore, we failed to conduct phylogenetic analysis to reveal the evolutionary relationship of Wg. Additionally, we identified another 12 and 9 unigenes from *L. migratoria* and *O. furnacalis* transcriptome, respectively (Table 1), which were potentially involved in the wing development, including homologs to *Drosophila apterous*, *engrailed*, *homothorax*, *teashirt*, *epidermal growth factor receptor*, *rhomboid*, *nubbin*, *pannier*, *notum*, *fat*, *four-jointed*, *dally-like*.

## Conclusions

In summary, we sequenced and characterized the transcriptome from the wing discs of *L. migratoria* nymph. The assembled sequence data comprising 91,907 unique transcripts provides a comprehensive sequence source for future *L. migratoria* study. We identified a large set of genes relevant to wing development with high significance, especially the genes involved in four signaling pathways – Notch, Hh, Dpp, and Wg signaling pathways, from *L. migratoria* transcriptome and another *O. furnacalis* transcriptome obtained previously. The explored wing development-related genes constitute an integrated picture of the development network, which provides the valuable clues for a better understanding of the wing development in *L. migratoria* and *O. furnacalis*. These development repertoire genes appear to be evolutionarily conserved to different extent. Functional analyses are necessary to verify our predictions. Nevertheless, the framework of information presented in this study should help to further understand the complex molecular mechanisms involved in wing development in *L. migratoria* and *O. furnacalis*, two insect species with different type of metamorphosis.

## Supporting Information

Figure S1 Assembled unigene length distribution of *L. migratoria* transcriptome. The *x*-axis indicates unigene size and the *y*-axis indicates the number (*left*) or percentage (*right*) of unigenes of each size.
(EPS)

Figure S2 Alignments of potential Meds in *L. migratoria*, *O. furnacalis* and *Drosophila*. Note: *L. migratoria* a26380 (green) and *O. furnacalis* Unigene4133 (purple) match well with the N-terminus of *Drosophila* Med (blue), while *L. migratoria* a11651 (black) and *O. furnacalis* Unigene17363 (red) are highly similar to the C-terminus of *Drosophila* Med (blue). The part without identity is shaded.
(EPS)

Figure S3 Schematic drawing of the Hh (A), Dpp (B), Notch (C), and Wg (D) signaling pathways. **(A)** In the presence of Hh, Hh binds to its receptor Ptc and Ptc relieves Smo repression. Smo accumulates and is activated by phosphorylation. This promotes its association with the complex including Costal 2 (Cos2), Fused (Fu), Suppressor of Fu (Su(Fu)) and Cubitus interruptus (Ci). Uncleaved Ci is released from the complex and acts as transcriptional activator in the nucleus and induces expression of target genes. **(B)** Dpp binds to its receptor complex including the type I receptor Tkv and type II receptor Punt which induces the phosphorylation of Tkv and in turn phosphorylates the signal transducer Mad. Phosphorylated Mad forms complex with Med and then translocates to the nucleus and induces expression of target genes. **(C)** Notch receptor is a transmembrane protein, with a large extracellular domain (NECD), a transmembrane domain and an intracellular domain (NICD). When its ligands Dl or Ser bind to the Notch, two proteolytic cleavage events are activated: the first cleavage is catalyzed by ADAM-family TACE metalloproteases; the second cleavage is mediated by γ-secretase which is an enzyme complex containing presenilin, nicastrin, PEN2 and APH1. The second cleavage liberates the NICD, which then migrates into the nucleus and cooperates with the DNA-binding protein CSL and its co-activator Mam to promote the transcription of downstream target genes. **(D)** Wg binds to the receptors Fz and Arr. The Wg-Arr-Fz complex binds to and activates the protein Dsh which then disrups the complex of APC/Axin/Sgg and results in an increase of free cytosolic Arm. Arm then translocates to the nucleus where it binds to transcription factor Pangolin (dTCF) and activates the expression of target genes.
(EPS)

Table S1 Primers for RT-PCR analysis.
(XLS)

Table S2 The statistics of annotated unigenes.
(DOC)
