# Supplementary material for: De Novo Transcriptome Analysis of Wing Development-Related Signaling Pathways in Locusta migratoria Manilensis and Ostrinia furnacalis (Guenée)
Source: PLoS One. 2014 Sep 10;9(9):e106770. doi: 10.1371/journal.pone.0106770 (PMC4160219; doi:10.1371/journal.pone.0106770)
Supplement: Table S2 — The statistics of annotated unigenes. (DOC) [file pone.0106770.s005.doc]

**Table S2. The statistics of annotated unigenes**

| Database | Nr | Nt | Swiss-Port | KEGG | COG | GO | All annotated | Not annotated |
| --- | --- | --- | --- | --- | --- | --- | --- | --- |
| Numbers of genes | 20,746 | 8,737 | 11,450 | 5,312 | 4,986 | 10,644 | 23,359 | 68,548 |
| Percentage (%) | 22.6 | 9.5 | 12.5 | 5.8 | 5.4 | 11.6 | 25.4 | 74.6 |

(Note: A total of 91,907 unigenes were obtained in *L. migratoria* transcriptome)
